# Health and Human Rights in Chin State, Western Burma: A Population-Based Assessment Using Multistaged Household Cluster Sampling

**DOI:** 10.1371/journal.pmed.1001007

**Published:** 2011-02-08

**Authors:** Richard Sollom, Adam K. Richards, Parveen Parmar, Luke C. Mullany, Salai Bawi Lian, Vincent Iacopino, Chris Beyrer

**Affiliations:** 1 Physicians for Human Rights, Cambridge, Massachusetts, United States of America; 2 University of California, Los Angeles, Department of General Internal Medicine and Health Services Research, Los Angeles, California, United States of America; 3 Global Health Access Program, California, United States of America; 4 Brigham and Women's Hospital, Boston, Massachusetts, United States of America; 5 Harvard Humanitarian Initiative, Cambridge, Massachusetts, United States of America; 6 Center for Public Health and Human Rights, Johns Hopkins Bloomberg School of Public Health, Baltimore, Maryland, United States of America; 7 Chin Human Rights Organization, Nepean, Ontario, Canada; 8 University of Minnesota Medical School, Minneapolis, Minnesota, United States of America; 9 Human Rights Center, University of California, Berkeley, Berkeley, California, United States of America; Institute of Tropical Medicine, Antwerp, Belgium

## Abstract

**Background:**

The Chin State of Burma (also known as Myanmar) is an isolated ethnic minority area with poor health outcomes and reports of food insecurity and human rights violations. We report on a population-based assessment of health and human rights in Chin State. We sought to quantify reported human rights violations in Chin State and associations between these reported violations and health status at the household level.

**Methods and Findings:**

Multistaged household cluster sampling was done. Heads of household were interviewed on demographics, access to health care, health status, food insecurity, forced displacement, forced labor, and other human rights violations during the preceding 12 months. Ratios of the prevalence of household hunger comparing exposed and unexposed to each reported violation were estimated using binomial regression, and 95% confidence intervals (CIs) were constructed. Multivariate models were done to adjust for possible confounders. Overall, 91.9% of households (95% CI 89.7%–94.1%) reported forced labor in the past 12 months. Forty-three percent of households met FANTA-2 (Food and Nutrition Technical Assistance II project) definitions for moderate to severe household hunger. Common violations reported were food theft, livestock theft or killing, forced displacement, beatings and torture, detentions, disappearances, and religious and ethnic persecution. Self reporting of multiple rights abuses was independently associated with household hunger.

**Conclusions:**

Our findings indicate widespread self-reports of human rights violations. The nature and extent of these violations may warrant investigation by the United Nations or International Criminal Court.

*Please see later in the article for the Editors' Summary*

## Abstract 2

Sollom and colleagues report the findings from a household survey study carried out in Western Burma; they report a high prevalence of human rights violations such as forced labor, food theft, forced displacement, beatings, and ethnic persecution.

## Editors' Summary

**Background:**

More than 60 years after the adoption of the Universal Declaration of Human Rights, thousands of people around the world are still deprived of their basic human rights—life, liberty, and security of person. In many countries, people live in fear of arbitrary arrest and detention, torture, forced labor, religious and ethnic persecution, forced displacement, and murder. In addition, ongoing conflicts and despotic governments deprive them of the ability to grow sufficient food (resulting in food insecurity) and deny them access to essential health care. In Burma, for example, the military junta, which seized power in 1962, frequently confiscates land unlawfully, demands forced labor, and uses violence against anyone who protests. Burma is also one of the world's poorest countries in terms of health indicators. Its average life expectancy is 54 years, its maternal mortality rate (380 deaths among women from pregnancy-related causes per 100,000 live births) is nearly ten times higher than that of neighboring Thailand, and its under-five death rate (122/1000 live births) is twice that of nearby countries. Moreover, nearly half of Burmese children under 5 are stunted, and a third of young children are underweight, indicators of malnutrition in a country that, on paper, has a food surplus.

**Why Was This Study Done?:**

Investigators are increasingly using population-based methods to quantify the associations between human rights violations and health outcomes. In eastern Burma, for example, population-based research has recently revealed a link between human rights violations and reduced access to maternal health-care services. In this study, the researchers undertake a population-based assessment of health and human rights in Chin State, an ethnic minority area in western Burma where multiple reports of human rights abuses have been documented and from which thousands of people have fled. In particular, the researchers investigate correlations between household hunger and household experiences of human rights violations—food security in Chin State is affected by periodic expansions of rat populations that devastate crop yields, by farmers being forced by the government to grow an inedible oil crop (jatropha), and by the Burmese military regularly stealing food and livestock.

**What Did the Researchers Do and Find?:**

Local surveyors questioned the heads of randomly selected households in Chin State about their household's access to health care and its health status, and about forced labor and other human rights violations experienced by the household during the preceding 12 months. They also asked three standard questions about food availability, the answers to which were combined to provide a measure of household hunger. Of the 621 households interviewed, 91.9% reported at least one episode of a household member being forced to work in the preceding 12 months. The Burmese military imposed two-thirds of these forced labor demands. Other human rights violations reported included beating or torture (14.8% of households), religious or ethnic persecutions (14.1% of households), and detention or imprisonment of a family member (5.9% of households). Forty-three percent of the households met the US Agency for International Development Food and Nutrition Technical Assistance (FANTA) definition for moderate to severe household hunger, and human rights violations related to food insecurity were common. For example, more than half the households were forced to give up food out of fear of violence. A statistical analysis of these data indicated that the prevalence of household hunger was 6.51 times higher in households that had experienced three food-related human rights violations than in households that had not experienced such violations.

**What Do These Findings Mean?:**

These findings quantify the extent to which the Chin ethnic minority in Burma is subjected to multiple human rights violations and indicate the geographical spread of these abuses. Importantly, they show that the health impacts of human rights violations in Chin State are substantial. In addition, they suggest that the indirect health outcomes of human rights violations probably dwarf the mortality from direct killings. Although this study has some limitations (for example, surveyors had to work in secret and it was not safe for them to collect biological samples that could have given a more accurate indication of the health status of households than questions alone), these findings should encourage the international community to intensify its efforts to reduce human rights violations in Burma.

**Additional Information:**

Please access these websites via the online version of this summary at http://dx.doi.org/10.1371/journal.pmed.1001007.

- The UN Universal Declaration of Human Rights is available in numerous languages
- The Burma Campaign UK and Human Rights Watch provide detailed information about human rights violations in Burma (in several languages)
- The World Health Organization provides information on health in Burma and on human rights (in several languages)
- The Mae Tao clinic also provides general information about Burma and its health services (including some information in Thai)
- A *PLoS Medicine* Research Article by Luke Mullany and colleagues provides data on human rights violations and maternal health in Burma
- The Chin Human Rights Organization is working to protect and promote the rights of the Chin people
- The Global Health Access Program (GHAP) provides information on health in Burma
- FANTA works to improve nutrition and global food security policies

## Introduction

**Figure 1:**
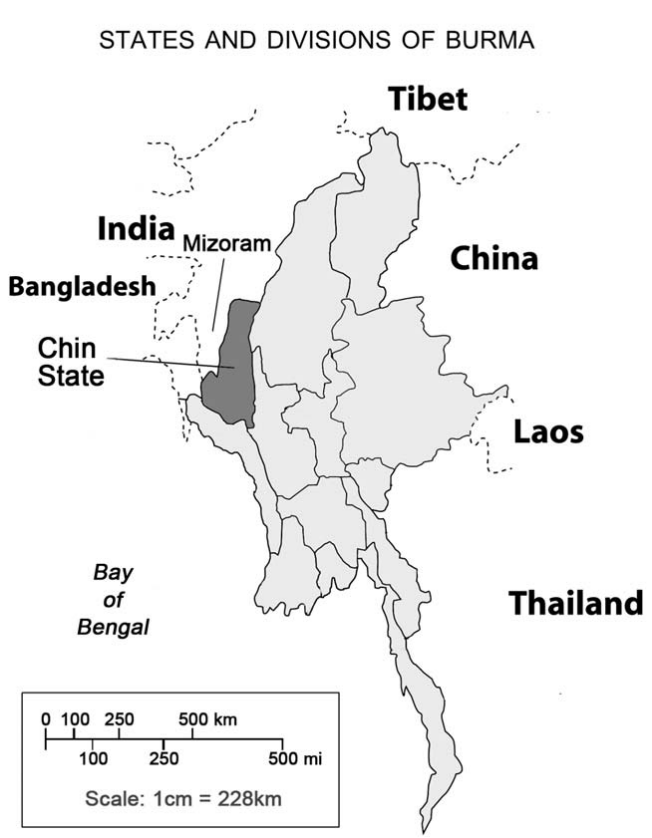
Map of Burma.

Investigators are increasingly using population-based methods to document human rights violations (HRVs) [1], i.e., abuses committed by state authorities of those rights and freedoms enshrined in various international treaties [2],[3]. Population-based survey research can generate quantitative measures of the prevalence of war-related sexual violence [4], genocide [5], and other conflict-related deaths [6],[7], refugee displacement [8], maternal mortality [9],[10], and discrimination against persons living with HIV/AIDS [11]. More recently, researchers have quantified the associations between HRVs and health outcomes [12],[13], including in eastern Burma [14],[15]. Few such data exist for western Burma, where ethnic and religious minority populations have poor health outcomes and lower socioeconomic status compared to central Burma and where human rights abuses have been reported [16]. Western Burma borders Bangladesh to the south and the Northeast Indian States of Mizoram, Manipur, and Nagaland to the north—remote regions that have been marked by insurgency, militarization, and allegations of human rights abuses (Figure 1). The UN Special Rapporteur on human rights for Myanmar, Tomás Ojea Quintana, reported in March 2010 that 75,000–100,000 undocumented Chins live in Mizoram State after having fled their homeland [16]. There has been little quantitative investigation of the forces driving this Chin exodus.

Though Burma is a food-surplus country [17] rich in natural resources [18], it ranks among the poorest in terms of health indicators. Burmese adults live 54 years on average, and the country's reported mortality rate for children under the age of 5 years (“under-5”) (122/1000 live births) is twice the rate of other countries in the region [19]. Over 40% of Burmese children under 5 are stunted, and one-third of children under 5 are underweight, suggesting high rates of acute and chronic malnutrition [20]. HIV/AIDS, malaria, and multidrug-resistant tuberculosis add to Burma's substantial burden of disease [21]. Despite this disease burden, the Burmese government allocated only 1.1% of total expenditures to health in 2005 (less than $1USD per capita) leading the WHO in part to rank Burma at the bottom (190/191) for health systems in the world [22].

The Chin and other ethnic minorities have suffered an array of reported human rights violations at the hands of successive military regimes, which have amplified these poor health outcomes [23],[24]. Five decades of military rule and the persecution of ethnic and religious groups has displaced more than 3.5 million civilians, making Burma the world's largest source of displaced persons [25]. Qualitative research has provided evidence that Burma's military junta may have committed crimes against humanity [16],[23],[26]–[28], defined as “the most serious crimes of concern to the international community” [29]. These crimes include murder, extermination, enslavement, deportation, imprisonment, torture, rape, group persecution, enforced disappearance, apartheid, and other inhumane acts, which are committed as part of a widespread or systematic attack against a civilian population [29]. Lacking, however, has been population-based research to assess the scale and scope of these alleged crimes among ethnic groups in western Burma. Through a population-based assessment, we sought to document reports of abuses against civilians in Chin State, the alleged perpetrators of such abuses, and whether these reported abuses are widespread.

Food security has been a longstanding concern in Burma. Chin State and surrounding areas have been particularly affected, with several factors implicated in worsening food security and malnutrition. The region has been burdened by a natural phenomenon known locally as the “rat famine” that occurs every 48 years. The rat famine is driven by a local species of bamboo (*Melocanna baccifera*) that flowers on a roughly half-century cycle and whose fruits dramatically increase rat populations. Once the enlarged rat population has eaten available wild foods, it overruns fields and can dramatically reduce crop yields [30]. The year 2009 was marked by a bamboo bloom and subsequent rat infestation, which increased malnutrition levels in multiple communities in the region [30]. A second factor in food insecurity has been the Burmese regime's focus on using farmland for cultivation of jatropha (*Jatropha curcas*, or physic nut, an inedible source of bio-fuel grown for export). Multiple reports of farmers being forced to switch from food crops to jatropha cultivation have been documented [31]–[33]. Finally, the Burmese military has a long history of appropriating food from villagers when moving through rural areas [23],[32],[34]; food security was therefore a primary focus of this investigation. We attempted to correlate measures of household hunger with household experience of reported human rights violations in the previous 12 months.

## Methods

This study was conducted by a collaborative group including researchers at Physicians for Human Rights, Center for Public Health and Human Rights at Johns Hopkins Bloomberg School of Public Health, The Global Health Access Program and several Chin health and human rights partner organizations.

Before the study from which these data are reported, exploratory qualitative work was undertaken, including interviews with 32 key informants from Chin civil society. The main study comprised a cross-sectional survey conducted by local surveyors (*n* = 22) whom we trained outside the country. Surveyors were evaluated daily on the content and application of survey material and methods; 22 of 23 local surveyors passed the 2-week training course. They then performed a multistage cluster sample survey of households in Chin State using a quantitative survey instrument and collecting anthropometric data to assess child malnutrition. The survey consisted of 87 questions among five domains of inquiry: household demographic data, access to health care, physical and mental health status, food insecurity, and human rights violations. These violations include acts of forced labor, pillaging, forced displacement, conscription of child soldiers, detention, disappearance, group persecution, murder, rape, and torture committed by government authorities or other armed forces.

We operationalized each reported human rights abuse, for example *forced labor* includes all nonvoluntary and nonremunerated work or service exacted from any person under menace of penalty or harm, and excludes compulsory military service, civic obligations, and minor communal services [35]. *Group persecution* refers to the intentional and severe deprivation of fundamental rights due to one's religious, ethnic, or other identity [29]. Torture is defined as the intentional infliction of severe physical or mental pain or suffering [36]. If such an incident reportedly lasted less than 10 minutes, we classified it as *beating*.

We employed strict inclusion criteria for each reported human rights violation to be included in our analysis. The respondent needed to: (1) answer a series of follow-up questions regarding each incident; (2) verify the reported incident occurred during the preceding 12 months; (3) identify the perpetrator of the incident as a government authority or member of another armed force—civilian or unknown perpetrators were excluded; and (4) affirm that s/he had personally experienced or was an eyewitness to the reported incident.

Data were collected during February and March 2010, and the period under investigation included events occurring 12 months before the survey research. This study was approved by the External Review Committee of Physicians for Human Rights, the institutional review board at the University of California Los Angeles Office for the Human Research Protection Program, and the Johns Hopkins Bloomberg School of Public Health Committee on Human Research.

### Sampling Frame

To create the sampling frame we compiled a complete list of 991 village names in Chin State from the UN-sponsored Myanmar Information Management Unit (MIMU) township maps [37] and the US Geographic Names Database with corresponding geographic coordinates [38]. We obtained ambient population estimates (the average population for a given location over 24 hours) for all rural villages from the 2005 Oak Ridge National Laboratory LandScan dataset [39] and used 2006 census data from the Union of Myanmar Ministry of Health for the nine urban centers [40]. (We used ambient population estimates since complete village-level census data were unavailable.) The total LandScan population estimate for Chin State (547,000) compared favorably with government-reported 2006 population figures (533,000) as well as with known village-level populations.

All nine townships within Chin State were included in the sample, which was stratified by urban and rural status. The number of clusters to be sampled was determined with the following approach: townships (T_i_) in Chin State were labeled T_1_, T_2_, T_3_ … T_9_. We listed population (P_i_) in each township as P_1_, P_2_, P_3_ … P_9_. ∑ P_i_  =  P, the total population in Chin State. We derived the number of clusters, X_i_, in each township, T_i_, where X_i_ = 90×P_i_/P, and ∑ X_i_ = 90.

For the second stage of the sample, a fraction of the total clusters X_i_ were assessed to be urban and the rest were considered rural. This determination was done by calculating township-specific urban-to-rural population ratios and then applying this ratio to the township-specific cluster count, X_i_. Lists of urban and rural villages were compiled by township, and the first cluster was selected using a random number generator. Subsequent clusters were selected by probability proportional to size (PPS) sampling.

For the third stage of cluster sampling, random start proximity sampling was used. In each urban and rural cluster, the surveyor assigned to that cluster walked the diameter (D) of the village and counted the number of visible houses on one side of the main road. The surveyor then returned to the center of the village (D/2), spun a pen, and headed in that direction. To minimize the potential bias of spin-the-pen methods [41], a household was chosen by randomly selecting a number from 1 to D/2. From this starting household, the surveyor proceeded to the closest adjacent residence until eight households were surveyed. A household was defined as a unit that ate together and had a separate entrance from the road.

### Informed Consent

Surveyors did not publicize their presence or the purpose of their visit when arriving in a village. Surveyors knocked on the door of the household selected by the cluster-sampling methods described above and asked for the adult head of the household (older than 18 years). Surveyors then informed the heads of household of the purpose of the survey, assured them that all information would be strictly confidential and that no names would be gathered, and that there would be no benefits or penalties for refusing or agreeing to participate. They were also informed that they did not have to answer any or all questions and that they could stop the interview at any time. Heads of household were interviewed about health and rights in the household over the previous 12 months, about their individual experiences, and about the health and nutrition status of any individual children or infants in the household. Consent was obtained verbally and marked *Y* or *N* on each paper survey. Separate informed consent was obtained from the head of household to collect anthropometric data among children 5–59 months of age.

### Statistical Analysis

Analysis of the survey data focused on reports of human rights violations, health outcomes, and the association between these variables. First, township-specific coverage and participation rates were estimated. The overall completion rate for the survey was defined as the total number of consenting households divided by the number of planned households (*n* = 720).

Second, the prevalence of household-level exposure to human rights violations was estimated for a variety of domains. These included (1) forced labor (any and type/task specific) and the reported responsible authority, (2) food security–related events (e.g., forced to give food, destruction of crops, theft or killing of livestock), (3) forced relocation or movement, (4) physical violence (e.g., death or injury by gunshot or landmine, beatings and torture, and sexual assault), (5) other violations including forced conscription, kidnapping, detainment, imprisonment, and religious or ethnic persecution. For each domain the overall percentage of affected households was estimated, and 95% confidence intervals (CIs) constructed.

Third, basic health outcomes were described. As per FANTA-2 (Food and Nutrition Technical Assistance II, USAID; http://www.fantaproject.org/) guidelines [42], household hunger was assessed by a scale that combined responses to three standard questions related to food availability. These included reports in the previous 30 days either of a complete lack of food, or of one or more household members going to sleep hungry or passing the entire day without food because of a lack of food in the household. Other health indicators included an assessment of general health and a two-question screen for depression using the Patient Health Questionnaire-2 (PHQ-2) [43],[44] (unpublished data). For each measure, the proportion of households affected was estimated, and 95% CIs constructed.

The fourth phase of analysis focused on estimating the association between each of the rights violations reported above and household hunger. For this analysis, moderate and severe household hunger (as measured by the household hunger scale) were combined. Households were stratified by this dichotomous variable (none/mild versus moderate/severe), and the proportion of households reporting exposure to each of the violations was compared. The ratio of the prevalence of household hunger comparing households exposed and unexposed to each violation was estimated using binomial regression with a log link function, and 95% CIs constructed. A multivariate model was constructed to examine the relationship between household hunger and a categorical variable combining exposure to three key food security-related violations: forced to give food, destruction of crops, and killing/theft of livestock. Prevalence rate ratios (PRRs), both crude and adjusted for household size, rural/urban locale, religion, report of forced labor, and possible impact of rats on crop yield, were estimated and 95% CIs constructed. In all analyses, relative differences in prevalence were modeled as ratios using binomial regression estimation with a log link function. When bivariate or multivariate models failed to converge, a Poisson distribution was used. Variance estimates for all prevalence estimates and for parameter estimates in the regression models were adjusted for the cluster-survey design using Taylor linearization. The statistical package STATA 11.0 was used for all analyses [45].

## Results

The study teams were able to conduct surveys in all nine townships in 100% of urban clusters and 98.7% of rural clusters (Table 1). At the household level, surveyors reached 100% of 112 urban households and 97% of 608 rural households. Overall participation was high, with 86.3% of heads of household consenting to participate, though there was some variation by township (Table 1).

**Table 1:** Summary of cluster and household coverage and participation.

Township	Urban						Rural						Total	
	Clusters			Households			Clusters			Households			Households	
	Intended	Reached	%	Intended	Reached	%	Intended	Reached	%	Intended	Reached	%	Consented	%
**Falam**	1	1	100	8	8	100	13	13	100	104	103	99	92	82.1
**Hakha**	3	3	100	24	24	100	6	6	100	48	48	100	56	77.8
**Thantlang**	1	1	100	8	8	100	10	10	100	80	79	99	86	97.7
**Kanpetlet**	1	1	100	8	8	100	2	2	100	16	16	100	24	100
**Matupi**	1	1	100	8	8	100	7	7	100	56	55	98	46	71.9
**Mindat**	2	2	100	16	16	100	6	6	100	48	48	100	61	95.3
**Paletwa**	2	2	100	16	16	100	14	14	100	112	105	94	101	78.9
**Tedim**	2	2	100	16	16	100	15	14	93	120	112	93	124	91.2
**Tonzang**	1	1	100	8	8	100	3	3	100	24	24	100	31	96.9
**Total (9)**	**14**	**14**	**100**	**112**	**112**	**100**	**76**	**75**	**98.7**	**608**	**590**	**97**	**621**	**86.3**

### Forced Labor

Surveyors asked respondents who had compelled them into forced labor during the previous 12 months. Households reported 1,570 separate incidents of forced labor. The majority (64.9%) of forced labor demands were reportedly imposed by the Burmese military, or Tatmadaw. The civilian representatives of the Tatmadaw, or Village Peace and Development Council (VPDC), were responsible for an additional 30.8% of all reported acts of forced labor. Burmese police and Chin ethnic forces were reportedly responsible for a minority of cases, at 2.3% and 1.9% respectively.

Household-level reporting of the prevalence of forced labor was high (Table 2). Overall, 91.9% of households (95% CI 89.7%–94.1%) reported at least one episode of an adult or child household member being subjected to forced labor in the 12 months before the interview (Table 2). More than three quarters of all households were forced to construct roads, buildings, or bridges (78.4%, 95% CI 72.2%–84.5%). Nearly 60% were forced to carry supplies for the armed forces (59.3%, 95% CI 51.5%–67.1%), and another 15% were forced to carry weapons (14.8%; 95% CI 8.4%–21.2%). A striking 77.4% (95% CI 70.8%–84%) of households were forced to grow jatropha (Table 2).

**Table 2:** Summary of reported human rights abuses, including food security–related and other violations.

Description	Households^a^	Cases	%	95% CI
**Human rights abuses**				
Household members killed	607	6	1.0	0–2.4
Household members tortured	609	23	3.8	2.1–5.5
Household members raped or sexually violated	603	17	2.8	1.3–4.4
Household members detained or imprisoned	609	36	5.9	2.0–9.9
Household members kidnapped or disappeared	607	29	4.8	1.6–7.9
Households that experienced religious/ethnic persecution	611	86	14.1	8–20.1
Household members suffering other inhumane acts	603	1	0.2	0–0.5
Other inhumane acts causing great suffering or serious injury: any forced labor	618	568	91.9	89.7–94.1
Forced to build roads, bridges, buildings	597	468	78.4	72.2–84.5
Forced to porter	602	357	59.3	51.5–67.1
Forced to carry weapons	567	84	14.8	8.4–21.2
Forced to cook or be a servant	567	105	18.5	12.6–24.5
Forced to sweep for landmines	567	8	1.4	0.0–2.8
Forced to grow jatropha or other crop	592	458	77.4	70.8–84
Forced to do other hard labor	557	90	16.2	10.3–22.0
**Food security–related violations/events**				
Forced to give food out of fear	601	304	50.6	42.4–58.7
Forced to provide money out of fear	605	259	42.8	35.2–50.5
Household crops/food stores stolen or destroyed	598	23	3.8	0.4–7.3
Home attacked or destroyed	607	29	4.8	1.5–8.1
Communal property attacked or destroyed	602	77	12.8	7.1–18.5
Household livestock stolen or killed	602	316	52.5	45.3–59.7
**Other violations/events**				
Households forced to move	602	74	12.3	8.2–16.3
Households forced to move to seek food	602	61	10	6–14.1
Children <15 years forcibly conscripted into armed forces	615	17	2.8	1.6–3.9
Adults forcibly conscripted into armed forces	615	35	5.7	1.4–10
Household members wounded from gunshot, explosion, or other deadly weapon	607	55	9.1	4.8–13.3
Household members beaten	609	68	11.2	7–15.3

Parameter estimates and their 95% confidence intervals are adjusted for the complex survey design.

a Total number of households responding to each respective question.

**Figure 2:**
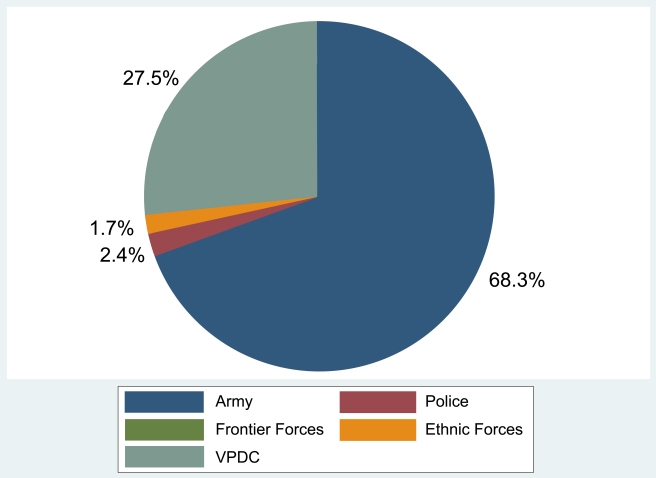
Proportion of reported crimes against humanity, by alleged perpetrator.

Households reported 2,951 incidents of abuse, including 1,768 of the most severe abuses that potentially meet criteria for crimes against humanity (Figure 2). All episodes of killing (*n* = 6), torture (*n* = 23), and rape or sexual violence (*n* = 17), as well as over 93% of imprisonment (*n* = 36), disappearance (*n* = 29), and ethnic or religious persecution (*n* = 86) reportedly were committed by the Burmese military. When considered together with forced labor (*n* = 568), the majority (68.3%) of these severe abuses reportedly were committed by the Burmese military. The civilian representatives of the Burmese military, the VPDC, reportedly were responsible for an additional 27.5% of severe abuses. Burmese police, border guards (NaSaKa), and Chin ethnic forces were responsible for a minority of reported incidents, at 2.4%, 0.1%, and 1.7%, respectively. A similar pattern was reported for 1,183 separate incidents of other human rights violations (unpublished data).

### Household-Level Prevalence of Other Severe Rights Violations

Direct physical violence included households reporting a member killed by gunshot or other deadly weapon, beatings, torture, and sexual violence. Beating or torture was the most commonly reported form of physical violence, reported by 14.8% of households (95% CI 10.1%–19.4%). Torture was reported by 3.8% of households (95% CI 2.1%–5.5%). Rape/sexual violence was the least common abuse, reported by some 2.8% of households (95% CI 1.3%–4.4%). Other reported violations included forced conscription of children under the age of 15 into the armed forces, reported by 2.8% of households (95% CI 1.6%–3.9%); kidnapping or disappearance of a household member, reported by 4.8% (95% CI 1.6%–7.9%); detention or imprisonment of a household member, reported by 5.9% (95% CI 2.0%–9.9%). Some 14.1% of households (95% CI 8%–20.1%) reported having experienced religious or ethnic persecution (Table 2).

Southern Chin state (Mindat, Paletwa, and Kanpetlet townships) had markedly higher densities of HRVs than other townships in the state. Rural areas have a higher density of HRVs than urban strata; and there were no reported HRVs occurring over the sampling frame period in the state capital, Hakha.

Rights violations related to food insecurity were common. More than half of all households were reportedly forced to give food out of fear of violence (50.6%, 95% CI 42.4%–58.7%), and 52.5% of household livestock were reportedly killed or stolen (95% CI 45.3%–59.7%) (Table 2). Forced displacement was reported by 12.3% of households, with a slightly smaller proportion reporting being forced to move to seek food (Table 2).

### Household Health

Household and individual measures of health included child malnutrition and a household hunger scale validated by FANTA-2. Individual-level perceptions of physical health were assessed for each interviewee. In all, the status of 158 children was assessed; 40.5% had some level of malnutrition, with 11.4% moderate (95% CI 5.0%–17.7%) and 13.3% severe (95% CI 7.2%–19.3%) (Table 3). Household-level hunger was also common, with 42.6% of households overall reporting moderate (29.8%; 95% CI 21.4%–38.1%) or severe (12.8%; 95% CI 7.6%–18.1%) hunger.

**Table 3:** Health indicators measured or reported among households in Chin State.

Individual Level Indicator	Children	Cases	%	95% CI
**Child malnutrition**				
None	158	94	59.5	50.3–68.6
Mild	158	25	15.8	9.2–22.5
Moderate	158	18	11.4	5.0–17.7
Severe	158	21	13.3	7.2–19.3
**Household/respondent level indicator**	**HH**	**Cases**	**%**	**95% CI**
**Household hunger (FANTA-2 definition)**				
None/mild	615	353	57.4	48.9–65.8
Moderate	615	183	29.8	21.4–38.1
Severe	615	79	12.8	7.6–18.1
**Perception of physical health in past 12 months**				
ery good/excellent	617	71	11.5	7.5–15.5
Good	617	220	35.7	30.6–40.8
Fair	617	148	24	18.6–29.4
Poor	617	178	28.8	23.3–34.4

### Household Hunger and Associations with Rights Violations

Bivariate analyses of associations between moderate to severe household hunger and forced labor demonstrated statistically significant associations of hunger with any forced labor, PRR 2.8 (95% CI 1.45–5.43) (Table 4). Large and statistically significant associations were also found between hunger and other rights violations, including being forced to give food out of fear, PRR 3.56 (95% CI 2.4–5.3); having household crops/food stores stolen or destroyed, PRR 1.91 (95% CI 1.37–2.67); and having livestock stolen or killed, PRR 2.55 (95% CI 1.84–3.54) (Table 4).

**Table 4:** Association between moderate/severe household hunger and human rights violations.

Description of Human Rights Violation	Total *N* a	Moderate/Severe HH Hunger			None/Mild HH Hunger			PRR	95% CI
		Exposed	Cases	%	Exposed	Cases	%		
**Physical violence**									
Household member(s) wounded/killed from gunshot, explosion or other	602	60	49	81.7	542	206	38	**2.15**	**1.67–2.77**
Household member(s) beaten or tortured	604	89	65	73	515	192	37.3	**1.96**	**1.56–2.46**
Household member(s) sexually assaulted or other inhumane act	598	18	12	66.7	580	240	41.4	**1.61**	**1.09–2.38**
**Food security–related violations/events**									
Forced to give food out of fear of violence	596	301	200	66.4	295	55	18.6	**3.56**	**2.4–5.3**
Forced to provide money	600	257	146	56.8	343	105	30.6	**1.86**	**1.36–2.53**
Household crops/food stores stolen or destroyed	594	23	18	78.3	571	234	41.0	**1.91**	**1.37–2.67**
Home attacked or destroyed	602	29	18	62.1	573	233	40.7	**1.53**	**1.05–2.22**
Communal property attacked or destroyed^b^	597	76	76	100	521	173	33.2	**3.01**	**2.42–3.75**
Household livestock stolen or killed	597	312	187	59.9	285	67	23.5	**2.55**	**1.84–3.54**
**Forced labor related violations/events**									
Any forced labor in the previous 12 months	614	564	253	44.9	50	8	16	**2.8**	**1.45–5.43**
Forced to build roads, bridges, buildings	584	465	229	49.2	119	23	19.3	**2.55**	**1.41–4.61**
Forced to porter	589	354	191	54.0	235	63	26.8	**2.01**	**1.37–2.95**
Forced to carry weapons	554	83	45	54.2	471	196	41.6	1.3	0.92–1.85
Forced to cook or be a servant	554	104	79	76	450	161	35.8	**2.12**	**1.63–2.76**
Forced to sweep for landmines^b^	554	8	8	100	546	232	42.5	**2.35**	**1.98–2.80**
Forced to grow jatropha or other crop	579	454	206	45.4	125	38	30.4	1.49	0.97–2.3
Forced to do other hard labor	544	90	42	46.7	454	195	43	1.09	0.74–1.59
**Other violations/events**									
Child or adult forced to serve in armed forces	610	50	37	74	560	222	39.6	**1.87**	**1.43–2.44**
Person(s) in household detained or imprisoned^b^	604	36	35	97.2	568	219	38.6	**2.52**	**2.08–3.06**
Person(s) in household kidnapped or disappeared	602	29	21	72.4	573	236	41.2	**1.76**	**1.31–2.36**
Household experienced religious/ethnic persecution	606	86	66	76.7	520	192	36.9	**2.08**	**1.62–2.67**
Household forced to move	601	73	44	60.3	528	206	39	**1.54**	**1.15–2.07**
Household forced to move to seek food	601	60	39	65	541	211	39	**1.67**	**1.25–2.23**

Parameter estimates and their 95% confidence intervals are adjusted for the complex survey design. Statistically significant associations are in **bold**.

a Total number of households responding to questions related to household hunger and the human rights violation specific to that row.

b Poisson distribution used to estimated relative prevalence rate as nearly all families reporting exposure to this violation also met the criteria for moderate/severe household hunger.

HH, household; PRR, prevalence rate ratio.

Because crop destruction by rats was a potentially important source of food insecurity not directly related to rights violations, we constructed a multivariate model to assess household hunger and rights violations adjusted for this factor, and for forced movement, forced labor, and household size. Having had no food security related violations was used as the reference, and we assessed the impact of one, two, and three reported violations on hunger (Table 5). This analysis showed a strong and consistent dose relationship between violations and hunger, with those households suffering three violations having a PRR of 6.51 (95% CI 3.11–13.64) for moderate to severe hunger (Table 5). Other, non-food security–related violations also remained independent predictors of household hunger, with those households having experienced two or more violations having roughly twice the rate of household hunger, PRR 1.87 (95% CI 1.31–2.68).

**Table 5:** Multivariate associations between moderate/severe household hunger and selected human rights violations, crude and adjusted for other covariates.

Outcome/Covariate	Unadjusted Associations		Adjusted Model 1		Adjusted Model 2	
	PRR	95% CI	AdjPRR	95% CI	AdjPRR	95% CI
**Number of food security violations** a						
One food security violation	**3.68**	**2.21–6.13**	**3.35**	**1.92–5.85**	**3.21**	**1.71–6**
Two food security violations	**5.2**	**3.18–8.52**	**4.53**	**2.59–7.9**	**3.83**	**2.07–7.07**
Three food security violations	**6.49**	**3.59–11.74**	**6.51**	**3.11–13.64**	**5.21**	**2.3–11.8**
**Number of physical violence violations** b						
One physical violence violation	**2**	**1.44–2.78**	—		1	0.74–1.35
Two or more physical violence violations	**2.46**	**1.8–3.36**	—		0.91	0.61–1.36
**Number of other violations/events** c						
One other violation/event	**2.11**	**1.54–2.9**	—		**1.61**	**1.21–2.13**
Two or more other violations/events	**2.47**	**1.83–3.35**	—		**1.87**	**1.31–2.68**
Reported forced to move			—		0.94	0.69–1.28
Reported forced labor			—		0.98	0.33–2.88
>50% crops destroyed by rats	**2.13**	**1.56 – 2.9**	**1.66**	**1.24–2.22**	**1.63**	**1.22–2.17**
**Household lives in rural area**			1.2	0.82–1.75	1.17	0.79–1.74
**Christian religion**			1.04	0.67–1.62	1.16	0.82–1.64
**Household size**			1.01	0.96–1.06	1	0.95–1.06

Model 1 adjusted for forced movement, forced labor, crop destruction by rats, rural, Christian religion and household size.

Model 2 adjusted for all covariates.

a Includes forced to give food, destruction of crops, and killing/theft of livestock.

b Includes wounded or killed from gunshot or explosion; beaten or tortured; and sexually assaulted or other inhumane act.

c Includes forced conscription of a child or adult; detained or imprisoned; kidnapped or disappeared [24]; and religious or ethnic persecution.

AdjPRR, adjusted prevalence rate ratio.

## Discussion

This population-based survey shows ongoing reports of human rights violations perpetrated by Burmese government authorities against the Chin ethnic minority in western Burma in 2010.

The prevalence of these alleged abuses, most notably forced labor at 91.9%, are exceptionally high, even for Burma [14],[15]. Although prevalences of other reported human rights violations may appear low in comparison (Table 2), we estimate a large number of households across Chin State have been affected. Based on these data, government authorities may have killed an estimated 1,008 household members, tortured 3,829 individuals, raped 2,821 adults and children, imprisoned 5,945 persons, disappeared 4,836 persons, and persecuted 14,207 households for their ethnicity or religion over the 12-month reporting period.

The lack of rights violations reported from the capital city, and the very high rates in rural areas, underscores the importance of population-based methods and broad survey coverage to identify human rights violations. The health impacts of these abuses have been marked, and suggest that indirect health outcomes of the abuses of the military regime likely dwarf the mortality from direct killings. While 61 individuals reportedly were wounded or killed from violence, and households that reported such a loss did have an increased likelihood of experiencing household hunger (Table 2), food-related and forced labor–related abuses were much more widely reported. Multiple violations of food security were independently associated with household hunger even after adjustment for ethnicity, religion, forced labor, household size, other violations, and—perhaps most importantly—reported crop destruction by rats, as shown in Table 5, Model 1. We conducted this analysis adjusted for rat destruction to attempt to assess what component of household hunger was independently associated with rights abuses. Clearly, households that were already marginal in terms of poverty or other sociodemographic variables (distance from markets, subminority ethnicity) would have been more vulnerable to hunger given exposure to these rights violations. We controlled for Christian faith in both Models 1 and 2 (Table 5) to adjust for this important risk for marginalization in this context, and violations of food security remained independently associated with hunger and demonstrated a dose–response relationship, with more violations leading to higher likelihood of household hunger. Nevertheless, there may have been some important variations across district or household levels for which we did not control.

The study was subject to several other limitations. Security concerns and the logistics of community-based sampling in this complex humanitarian context meant that biological samples could not be collected. In previous, related surveys by our group, measures such as malaria parasitemia have proved to be a robust correlate of health status and access to health care [14]. Middle–upper arm circumference measures were collected, but findings related to nutritional status of children aged 5 and under should be interpreted with caution because of low participation. Participation was low because many children were not in the household at the time of the survey, and security concerns meant that timely searches for all children in the household could not safely be done. The interviews were self-reports from heads of household over a 12-month recall period, so data are subject to recall bias. Specific probes were used when rights violations were reported to try and verify the time sequence of events, but absolute verification of the 12-month period prior to the interview was not possible. Heads of household were told that the survey contained questions on food security and on human rights violations, so there is the potential that the consent process itself may have biased the results in favor of reporting abuses; however, fear of reporting abuses, the use of strict inclusion criteria for each reported human rights violation, and the stigma associated with certain abuses such as rape may cause under-reporting of such abuses. The very consistent reporting of specific abuses, including forced labor and forced jatropha cultivation, both at much higher rates than in other related studies using the same consent approach, suggests that this bias is modest at best, and that these abuses are strikingly common in the Chin area.

The UN Special Rapporteur for human rights in Myanmar and others point to an abundance of qualitative research as evidence of crimes against humanity across Burma [16],[23]. We note that at least eight of the violations (murder, torture, rape, imprisonment, enforced disappearance, group persecution, forced displacement, forced labor) that we surveyed fall within the purview of the International Criminal Court (ICC) [29]. However, for the ICC to establish whether crimes against humanity have been committed, three common elements to such crimes generally must be established: (1) prohibited acts (“attacks”) took place after 1 July 2002 when the ICC treaty entered into force; (2) such prohibited acts were committed as part of a widespread or systematic attack directed against a civilian population; and (3) the perpetrator intended or knew that the conduct was part of the attack [46]. All reported human rights violations in our study occurred during the immediate 12 months before the interview in 2010 and thus fall within the temporal jurisdiction of the ICC. Additionally, our data show that the 1,768 attacks were directed against a relatively large body of civilian victims. Among our sample of 621 households representing 3,281 individuals, 49.9% are male, and 50.1% are female. The mean age is 25.5 years (95% CI 24.6–26.3 years) ranging from 0 to 98 years with the following proportions: <5 years 10.6%, <15 years 35.5%, and >65 years 3.5%. Although it is possible that the remaining 10.9% (comprising men aged 15–65) included noncivilian armed actors, it is unlikely that targeting of armed groups could account for our findings, as there is currently no active armed conflict between ethnic armed forces and the Burmese military in Chin State. We surveyed households throughout Chin State in both rural and urban areas. Data on self-reports of human rights violations reveal that the 1,768 alleged attacks took place in all of Chin State's nine townships. Although there is no threshold definition of what constitutes “widespread,” these data provide evidence that these reported abuses occurred on a large scale with numerous victims (Table 2). However, our study does not address the third element of culpability related to perpetrator intent. Thus, further evidence would be needed to establish the third element of individual culpability for these abuses and this evidence would likely stem from a UN commission of inquiry or formal ICC investigation.

## Abbreviations

CI: confidence interval
HRV: human rights violation
ICC: International Criminal Court
PRR: prevalence rate ratio
VPDC: Village Peace and Development Council
